# Digital Tinnitus Counseling in Clinical Practice: A Multicenter Randomized Controlled Trial

**DOI:** 10.3390/audiolres15060173

**Published:** 2025-12-09

**Authors:** Petra Brueggemann, Gernot G. Supp, Paul Schmidt, Birgit Mazurek

**Affiliations:** 1 Tinnitus Center, Charité–Universitätsmedizin Berlin, 10117 Berlin, Germany; petra.brueggemann@charite.de; 2 Delta Science SL, 38632 Arona, Santa Cruz de Tenerife, Spain; gernot@delta-science.eu; 3 Statistical Consulting for Science and Research, 13086 Berlin, Germany; paul.schmidt.mail@gmail.com

**Keywords:** tinnitus counseling, digital health, eHealth, DiGA, smartphone, mobile app, distress, daily burden, coping, management

## Abstract

**Background/Objectives**: Subjective tinnitus, defined as the perception of sound without an external source, is a common and often debilitating condition. In the absence of pharmacotherapy, disease management guidelines recommend counseling interventions to alleviate tinnitus-related distress and improve patient outcome. This study evaluated the benefit of guideline-compliant counseling provided by “Meine Tinnitus App”, a smartphone-based application, for the treatment of subjective tinnitus. **Methods**: A randomized controlled study was conducted in 204 patients with confirmed chronic subjective tinnitus enrolled at 33 ear, nose and throat (ENT) practices in Germany. Tinnitus improvement was evaluated after 10 weeks of intervention (digital counseling in addition to standard care vs. standard care only). The primary endpoint was the change in tinnitus-related distress (measured by the Mini-TQ-12 validated questionnaire). The secondary endpoint was the change in tinnitus-associated daily burden and coping difficulties (measured by the validated BVB-2000 questionnaire). Treatment effects for the primary and secondary endpoints were represented by the estimated marginal means (EMMs). **Results**: Patients of the intervention group showed a significant reduction in tinnitus-related distress (EMM [95% CI]: 4.5 [3.3–5.8]; *p* < 0.001) and a significant improvement in tinnitus-associated daily burden and coping difficulties (EMM [95% CI]: 0.5 [0.2–0.7]; *p* < 0.001) compared to patients of the control group, with large to moderate effect sizes (Hedges’ g between 1.1. and 0.5). These positive treatment effects were confirmed by responder and sensitivity analyses. Additionally, patients with high vs. low app usage showed a greater improvement in treatment effect for both endpoints (*p* < 0.05), further supporting the health benefits of digital counseling. **Conclusions**: This study demonstrated the efficacy of tinnitus counseling provided by “Meine Tinnitus App” to alleviate tinnitus-related distress, daily burden, and coping difficulties in patients with subjective tinnitus (German Clinical Trials Register DRKS00025379).

## 1. Introduction

Tinnitus is defined as the perception of sound in the absence of an acoustic stimulus [1,2,3,4,5,6]. It is a common condition, affecting up to 30% of the global adult population [2,4,7,8]. Ten to 15% of affected people experience prolonged (i.e., chronic) tinnitus requiring medical attention [2,4,7,8,9]. Tinnitus’ prevalence increases with age [4,6,7,8,9] and has progressed in recent decades [10]. Tinnitus is most frequently subjective, i.e., perceived only by the patient [3,4]. Subjective chronic tinnitus is often associated with co-morbidities, such as hearing loss, emotional distress, sleep disturbance, and attention and concentration deficits, resulting in a significant impairment of quality of life and an increased risk of anxiety and depression [4,11,12]. Conversely, evidence points to a bidirectional relationship between tinnitus-related distress and depression severity [13]. While the mechanisms underlying this association remain under investigation, the serotonin hypothesis proposes that reduced central serotonin levels influence the pathophysiology of depression and may also affect the perception or severity of tinnitus [14,15,16].

Subjective tinnitus is a heterogeneous disorder and its treatment is challenging, especially in the absence of an identified underlying cause. In the chronic stadium, no specific curative pharmacotherapy exists and treatment options are limited [5,6,17,18,19,20,21,22,23]. National and international guidelines for tinnitus management underline the importance of a thorough assessment of tinnitus severity (using validated tinnitus-specific questionnaires) and the benefit of counseling and cognitive behavior therapy (CBT) to alleviate the severity of tinnitus symptoms and reduce their negative impact on the patient’s life [5,8,24,25,26,27,28,29].

Tinnitus counseling is considered the most important component in tinnitus management [5,30]. This psycho-educational approach aims to provide information and advice to patients to help them understand their tinnitus, guide them into habituation to the perception of their tinnitus and help them cope with symptoms and their consequences on daily life (insomnia, emotional distress, attention and concentration disorders) [18,30]. Despite official recommendations, evidence for the efficacy of tinnitus counseling is surprisingly sparse, highlighting a clinical gap [22].

Beside this clinical gap, patient access to tinnitus counseling can be challenging and varies between countries. Barriers to proper tinnitus counseling may include a lack of specialty clinics or teams, a lack of psychology knowledge of healthcare providers, a lack of time for counseling, difficulty related to payment for services, and variations in available treatment options [5,6,31,32]. These barriers especially impact help-seeking tinnitus sufferers co-experiencing mental health disorders, who are in urgent need of specialized and effective medical support [31,33].

In this context, a plethora of tinnitus self-management tools such as mobile applications (“apps”) have emerged, most of which have not been validated, neither for their efficacy (in randomized clinical trials) nor for their safety and suitability for use according to regulations (e.g., CE-marking in the European Union) [5,34,35,36,37,38]. So far, a few randomized controlled trials have investigated the potential benefit of tinnitus apps that include elements of CBT and/or of counseling (in the form of information and education) [39,40,41,42,43,44,45]. While the availability and format of smartphone-based therapeutic interventions are appealing to users, their lack of clinical and regulatory validation leaves patients with uncertainty and no reliable options.

In this tinnitus app landscape, the smartphone-based “Meine Tinnitus App” (German for “My Tinnitus App”) offers guideline-compliant counseling therapy to patients with chronic subjective tinnitus. “Meine Tinnitus App” is a CE-marked app developed within the German Digital Health Application (DiGA) framework and eHealth infrastructure. It was granted reimbursement by statutory health insurance in Germany and is thus free to use for tinnitus patients upon prescription by their medical practitioner.

The present two-arm randomized controlled study evaluated the benefit of guideline-compliant counseling provided by “Meine Tinnitus App” in patients with confirmed chronic subjective tinnitus receiving standard care from their ear, nose and throat (ENT) practitioner (intervention group; n = 101) vs. tinnitus patients under standard care only (control group; n = 101). The primary endpoint was the change in tinnitus-related distress, from baseline to 10 weeks of app intervention vs. control, as measured by the Mini-TQ-12 questionnaire. The secondary endpoint was the change in tinnitus-associated daily burden and coping difficulties, from baseline to 10 weeks of app intervention vs. control, as measured by the BVB-2000 questionnaire. The combined use of Mini-TQ-12 and BVB-2000 in this study supports efficient measurement of tinnitus-related distress, comparability with widely used international questionnaires (THI, TFI, TQ), and robust assessment of change in both tinnitus distress and broader psychosocial adaptation—thus addressing clinical interpretability and international comparability of outcome measures [46,47,48,49,50,51]. This study thus tested two confirmatory hypotheses: (i) tinnitus-related distress would improve significantly more in the intervention group than in the control group, and (ii) tinnitus-associated daily burden and coping difficulties would decrease significantly more in the intervention group than in the control group.

## 2. Materials and Methods

### 2.1. Participants and Study Design

#### 2.1.1. Description of Participants and Centers

This prospective, multicenter, randomized controlled study was conducted between 20 July 2022 (first patient in) and 12 April 2023 (last patient out) in 204 tinnitus patients recruited from 33 ENT centers located in five federal states in Germany (Schleswig-Holstein, Mecklenburg-Western Pomerania, Hamburg, Lower Saxony and North Rhine-Westphalia). The intervention group received standard tinnitus care (typically, one consultation session with an ENT physician) in combination with digital tinnitus counseling using the “Meine Tinnitus App” (see Section 2.2). The control group received standard tinnitus care without digital treatment. The app-based intervention design did not allow for blinding.

#### 2.1.2. Patients’ Recruitment and Randomization

To be included in the study, patients with confirmed subjective tinnitus (i.e., diagnosed by their ENT doctor) should be ≥18 years of age and should have tinnitus for ≥3 months, completed hearing aid fitting if indicated, sufficient reading and writing skills, and sufficient knowledge of German. Exclusion criteria were the existence of acute or chronic mental illness or disorder (including depression, anxiety and suicidality), tinnitus-specific psychological treatment within the last 2 years, and previous use of Tinnitracks and/or Meine Tinnitus App mobile applications (Sonormed GmbH, Hamburg, Germany; see Section 2.2). Patients’ recruitment was conducted by ENT physicians based on the study inclusion and exclusion criteria. Block randomization (block of six patients, three for intervention and three for control, randomly assigned to either arm) was operated at recruitment by the ENT practitioners, to ensure balanced group sizes between the intervention and control groups.

#### 2.1.3. Study Endpoints

The primary endpoint of the study was tinnitus-related distress, as measured by the total score of the Mini-TQ-12 questionnaire [52,53] in the intervention and control groups, at study inclusion (t0) and after 10 weeks of study duration (t1) (see Section 2.3.1). The secondary endpoint of the study was the change in daily burden and coping difficulties associated with tinnitus, as measured by the total score of the BVB-2000 questionnaire [46,47] in the intervention and control groups, at study inclusion (t0) and after 10 weeks (t1) (see Section 2.3.2). The first data collection (t0) took place in the ENT practice, directly after inclusion in the study. The second data collection (t1) took place as a postal survey. To improve data quality, study nurses followed up each participant per telephone call, once within 2 weeks of study inclusion, to answer questions the participant might have and add missing content to the t0 questionnaires, if any. After the t1 questionnaires were sent by post, the study nurse may call again the participant to add missing content or if the questionnaires were not returned after 14 days.

#### 2.1.4. Ethics Statement

This study was conducted in accordance with the Declaration of Helsinki and approved by the Ethics Committee of the Medical Association of Westphalia-Lippe and the Westphalian Wilhelm University, Germany (registration number AZ-2021-325-fs, dated 18 June 2021). The study was registered at the German Clinical Trials Register (DRKS) under number DRKS00025379 on 12 July 2021. Informed consent was obtained from all subjects involved in the study, on the day of recruitment and prior to the first data collection (t0).

#### 2.1.5. Ethical Data Handling and GDPR Compliance

All data collected during digital intervention were handled in strict accordance with the principles of the EU General Data Protection Regulation (GDPR, EU 2016/679). Participants provided informed consent prior to data collection, and all personal data were anonymized or pseudonymized to ensure confidentiality. Data storage and processing were conducted on secure, encrypted servers, with access restricted to authorized research personnel only. The study protocol was reviewed and approved by the appropriate institutional ethics committee, and participants retained the right to withdraw their data at any time without consequence.

### 2.2. Smartphone-Guided Tinnitus Counseling Using “Meine Tinnitus App” (MTA)

“Meine Tinnitus App” (German for “My Tinnitus App”; Sonormed GmbH, Hamburg, Germany) is a CE-marked native iOS and Android smartphone application (“app”) intended to provide direct support to patients with confirmed subjective tinnitus (WHO’s international classification ICD-10 code H93.1) in the form of guideline-compliant counseling therapy (https://www.meinetinnitusapp.de; accessed on 4 November 2025). The app was developed as part of the German Digital Health Application (DiGA) framework and approved by the Federal Institute for Drugs and Medical Devices (BfArM), which certifies the safety, functionality, quality, data protection and data security of low-risk and low-threshold medical devices. In this context, “Meine Tinnitus App” (thereafter referred to as “MTA”) was granted reimbursement by statutory health insurance in Germany. It can thus be prescribed by medical practitioners and is free to use for tinnitus patients.

MTA empowers patients to manage their tinnitus, taking proactive steps to improve their health. It aims to reduce patient’s tinnitus-related distress, increase resilience and reduce impairment in everyday life. The app is grounded in the tinnitus counseling method and delivers structured psychoeducational content that incorporates cognitive behavioral therapy (CBT) principles. It provides guidance on tinnitus self-management through comprehensive tinnitus-specific multimedia content, including: app instructions, understanding tinnitus and its possible causes, understanding the biology of hearing, of tinnitus and of associated hearing loss problems, describing tinnitus perception and tinnitus-induced psychological stress, understanding and controlling attention, improving concentration, controlling thoughts, learning to deal with stress, learning to sleep peacefully, preventing relapse.

Topics are organized in lessons, tailored to address each user’s most pressing issues (such as emotional stress, concentration deficits, hearing problems, or sleep disorders), based on initial app-based survey results [54,55]. A lesson consists of several units optimized for smartphone use, to be completed step-by-step by the patient. At the end of each lesson, a quiz-style game helps review the material and enhance the patient’s motivation. Additionally, the patient’s learning progress is tracked, and feedback on acquired knowledge and skill levels is provided. Therapy adherence is further encouraged through interactive motivational elements (e.g., graphics, video, audio and text elements, game points to display progress, reminder and notification functions).

The use of MTA was designed to be easily integrated into the user’s daily routine. Patients decide when and for how long they engage with the content, as lessons can be paused at any time. It is advised to complete one lesson per week (each lasting 60–90 min). According to this schedule, the initial use of the app will span over 10 weeks (10 lessons, following a welcome and an introduction module). After completing a lesson, patients are encouraged to continue working on the strategies and exercises taught, to reinforce their integration into everyday life and prevent relapse. The app’s accessibility features and individualized pacing further support engagement across diverse patient populations, distinguishing it from other tinnitus apps that may lack structured therapeutic content.

Of note, the sum of all completed units (mandatory units and bonus units) served as a measure of app usage in a subgroup analysis conducted in patients of the intervention group (see Section 2.5.7).

### 2.3. Assessment of Tinnitus-Related Distress, and Tinnitus-Related Daily Burden and Coping Difficulties

#### 2.3.1. Mini-TQ-12 Scoring (Primary Endpoint)

To assess tinnitus-related distress, a standardized and validated questionnaire with 12 items, the so-called Mini-TQ-12 (“Mini-TF-12” in German) [52,53] was used. The Mini-TQ-12 questionnaire is derived from the longer tinnitus questionnaire (TQ) by Goebel and Hiller [56], a well-established survey of tinnitus-related distress in German-speaking regions [5,8,24,25,26], itself translated and adapted from the original questionnaire developed by Hallam et al. [57]. The 12 items of the Mini-TQ-12 capture several dimensions of tinnitus burden: emotion, cognition, tension, psychosocial distress, sleep disturbance, and concentration disturbance. The Mini-TQ-12 shows good test–retest reliability (Pearson correlation, r = 0.89) and a very high correlation with the total score of the original TQ (Pearson correlation, r = 0.92) [52,53]. The TQ and Mini-TQ-12 are well validated and widely used to assess and classify tinnitus severity, including in the context of therapeutic intervention in several clinical studies [48,58,59,60]. The Mini-TQ-12 questionnaire is available online in its original German version [61].

#### 2.3.2. BVB-2000 Scoring (Secondary Endpoint)

To assess burden and coping difficulties associated with tinnitus in everyday life, the so-called BVB-2000 questionnaire (“Bochumer Veränderungsbogen-2000” in German or “Bochum Change Questionnaire”) was used [46,47]. The BVB-2000 is an open-source standardized and validated self-assessment questionnaire derived from the well-acknowledged VEV (“Veränderungsfragebogen des Erlebens und Verhaltens”) questionnaire by Zielke and Kopf-Mehnert [62], which assesses perceived changes in experiencing and behavior. The BVB-2000 has been validated as an independent instrument to measure changes in well-being and behavior, covering dimensions of psychological stress common to all disorders: relaxation/tension, mood, self-esteem, social interaction, meaning, and ability to act [46,47,63,64]. The questionnaire is commonly used in psychotherapy to evaluate the success or the course of therapy [63,64]. It is also commonly used as a ‘single-point measurement’ at a given time point to capture a perceived change (improvement or deterioration) in experience or behavior relative to a defined reference time point [47,64].

#### 2.3.3. Contextualization

Both Mini-TQ-12 and BVB-2000 interplay with other well-established tinnitus questionnaires: the THI (a widely used 25-item questionnaire assessing functional, emotional, and catastrophic reactions to tinnitus) and the TFI (designed to capture functional impact of tinnitus, responsiveness to change, and international applicability). Studies show the THI correlates with TQ and its short forms at r ≈ 0.57–0.70 for baseline severity categories across languages [49,51]. The Mini-TQ-12’s strong correlation with the full TQ suggests that changes detected by it are likely to reflect changes that would also manifest in the THI or TFI, albeit with less burden. As such, using the Mini-TQ-12 (overall score) allows efficient benchmarking against standard instruments (THI, TFI) while retaining sensitivity and comparability. The addition of BVB-2000 adds a dimension of change measurement in psychosocial functioning, complementing the tinnitus-specific distress focus.

### 2.4. Additional Baseline Assessments

General health literacy was assessed at baseline (t0) using the standardized and validated 16-item HLS-EU-Q16 questionnaire. Four categories of health literacy were defined based on the total score: inadequate, problematic, sufficient and excellent [65]. Several tinnitus-related characteristics were also assessed by questionnaire at baseline, including tinnitus perception (tonal/atonal), hearing problems, use of hearing aids, sensitivity to noise, number of tinnitus treatments, tinnitus loudness, tinnitus annoyance, speech comprehension problems, medication intake for tinnitus, and other forms of tinnitus therapy.

### 2.5. Statistical Analysis

Analyses were conducted on the full analysis set (FAS), defined as all patients with baseline (t0) data for both the primary and secondary endpoints. Data analysts were not blinded to patients’ group allocation. However, all analyses were conducted according to the predefined statistical analysis plan (SAP). Statistical analyses were performed within R version 4.2.2 (R Core Team 2021; [66]).

#### 2.5.1. Sample Size Estimation

Sample size estimation was determined assuming a directed hypothesis, equal group sizes, an effect size of 0.5, a significance level of α = 0.025 and a probability of type 2 error of 0.8. In addition, it was assumed that due to the primary imputation method for missing values, the effect size should be reduced by 20% and thus should lie at d = 0.4. This resulted in a minimum total sample size of 200 study participants (n = 100 participants per group).

#### 2.5.2. Handling of Missing Values

Missing values at endline (t1) for the different analyses were handled as follows. For the change from t0 to t1 analyses of the primary and secondary endpoints (see Section 2.5.4), missing values were handled using the reference-based multiple imputation approach, combining the jump-to-reference (with reference defined as the control group) method of Carpenter et al. [67] with a bootstrap estimate for the variance of the treatment effect [68,69]. One thousand (1000) iterations were performed for bootstrapping, and 5 imputed datasets were generated for each bootstrap iteration.

For the single-point measurement analysis of the secondary endpoint (BVB-2000; see Section 2.5.4), missing values were handled using the predictive mean matching procedure [70]. The number of candidates for imputation was set to 5 and 100 imputed datasets were generated each. A potential influence of the imputation methods on the results was tested in sensitivity analyses in the subgroup of patients with a complete t0 and t1 dataset (i.e., without imputation of missing values; see Section 2.5.6)

Missing data that were subsequently collected per telephone follow-up by the study nurses were recorded with a corresponding ID and the date of follow-up. A potential influence of the study nurse factor in data evaluation was tested using a sensitivity analysis in patients without intervention of a study nurse follow-up (see Section 2.5.6).

#### 2.5.3. Group Comparison Testing

Differences between control and intervention groups were tested using the two-sample *t*-test assuming unequal variances (Welch’s test) for continuous variables, the chi-square (χ^2^) test for categorical variables or Fisher’s exact test in case of <5 entries.

To avoid an increase in Type I error due to testing group differences in the two endpoints, the family-wise error rate (FWER) was controlled using a serial Gatekeeping test strategy [71,72]. Thus, the secondary endpoint was only analyzed if the analysis of the primary endpoint showed a statistically significant group difference at the significance level of 0.05. The significance level for the group comparison of the secondary endpoint was also set at 0.05. This strategy ensured an overall FWER ≤ 0.05.

#### 2.5.4. Assessment of Treatment Effect from Baseline (t0) to Endline (t1)

Two methods were employed to evaluate the treatment effect in the intervention and control groups: (i) the change from baseline according to the difference in evaluation at t0 and t1 using the Mini-TQ-12 and BVB-2000 questionnaires (primary and secondary endpoints), and (ii) the single-point measurement at t1 of the BVB-2000 questionnaire (secondary endpoint).

The change between baseline (t0) and endline (t1) score values for the primary and secondary endpoint analyses was calculated using the following formula, so that improvement was expressed in positive values and deterioration in negative values:

Change in the Mini-TQ-12 for patient i:

Δ(Mini-TQ-12)_i_ = Mini-TQ-12_t0i_ − Mini-TQ-12_t1i_

Change in the BVB-2000 for patient i:

Δ(BVB-2000)_i_ = BVB-2000_t1i_ − BVB-2000_t0i_

For each endpoint, differences in change from baseline to endline in the control and intervention groups were tested using a linear mixed regression model [73], and expressed by the estimated marginal means (EMM) [74]. Baseline score (Mini-TQ-12/BVB-2000) and age (in years) were considered as continuous covariates, sex (male/female) was considered as a categorical covariate. Estimation of unknown parameters was carried out using the restricted maximum likelihood (REML) criterion. The model assumed a normal distribution of the conditional distribution of the endpoints. Since the endpoints are differences, deviations from this assumption at the lower and upper ends of these difference distributions are possible. Accordingly, a sensitivity analysis assumed the Student-t distribution as the conditional distribution for the endpoints (see Section 2.5.6).

The so-called ‘single-point measurement’ feature of the BVB-2000 (see Section 2.3.2) was also used to measure the change in daily burden and coping difficulties associated with tinnitus. Practically, participants were asked through the BVB-2000 at t1 to what extent an improvement/deterioration had occurred since baseline (t0, defined as reference time point). Results from the ‘single-point measurement’ analysis were compared to those of the secondary endpoint analysis based on the difference in change from t0 to t1 described above.

Treatment effects for the primary and secondary endpoints (i.e., treatment difference between the intervention and control groups) were represented by the difference in EMM between both groups and the corresponding two-sided 95% confidence intervals (CI). For EMM estimation assuming a normal distribution, *p*-values were based on the directed hypothesis of an improvement in the intervention group and were thus one-sided. In the case of full Bayesian estimation (assuming a Student-t distribution; see Section 2.5.6), the point estimates of the EMM are represented by the posterior median and the 95% CI by the highest posterior density intervals. The posterior probabilities for superiority of the intervention group over the control group were used as *p*-values. Accordingly, a high *p*-value was associated with a significant treatment effect.

#### 2.5.5. Practical Relevance Testing

The assessment of practical relevance was carried out using standardized mean differences (Hedges’ g) and responder analyses.

For the standardized mean differences, Hedges’ g was calculated, taking into account the adjustment for the specified covariates and the different ENT practices [75]. A Hedges’ g value of 0.2 was used as the irrelevance threshold. If the lower limit of the CI exceeded this value, the effect was considered as relevant. Hedges’ g measures of effect size were interpreted as follows: a Hedges’ g of 0.2–0.5 (0.2 ≤ g < 0.5) was interpreted as a small effect size (small difference between groups); a Hedges’ g of 0.5–0.8 (0.5 ≤ g < 0.8) was interpreted as a medium effect size (moderate difference between groups); a Hedges’ g of 0.8 and higher (g ≥ 0.8) was interpreted as a large effect size (substantial difference between groups) [76].

To evaluate the clinical relevance of the intervention beyond the significance assessment, we also conducted responder analyses [77]. For these responder analyses, response criteria were defined according to standard classifications widely used in medical practice. For the Mini-TQ-12, four standard tinnitus severity levels were considered: “mild” (score 0–7), “moderate” (score 8–12), “severe” (score 13–18), and “extremely severe” (score 19–24) [52,53]. As response criterion, an improvement by at least one level within this classification was chosen. For the BVB-2000, two responder categories were considered: “worsened” (score < 3.36) and “improved” (score > 4.4) [47,64]. Based on these categories and on the ‘single-point measurement for direct change’ feature of the BVB-2000 questionnaire, a BVB-2000 score > 4.4 (“improved”) at t1 was chosen as response criterion. For both endpoints, the relative risk (RR) for a non-response was calculated so that the superiority of the intervention group was shown as a value < 1.0. Respective two-sided 95% CI were calculated according to the Wald’s method based on maximum likelihood estimation [78]. In addition, an adjusted variant of the RR was calculated to control for the specified covariates (baseline score value, age and sex) using mixed regression models for binomially distributed target variables and logit link functions [79]. The adjusted relative risk (ARR) and the corresponding 95% CI were calculated by back-transforming the estimated marginal risks using the natural logarithm. A value of 1.0 was used as the irrelevance threshold for the RR. If the lower limit of the CI exceeded this value, the effect was considered as relevant.

#### 2.5.6. Sensitivity Analyses

The robustness of the data was tested using sensitivity analyses.

A first sensitivity analysis assessed the validity of the imputation method by repeating the primary and secondary endpoint analyses without imputation procedures (i.e., in patients with valid endpoints values at both t0 and t1).

The second sensitivity analysis assessed the validity of the distributional assumption of the regression models. To counter against possible departures from normality and reduce the influence of potential outliers, we repeated the primary and secondary endpoint analyses with a model utilizing Student’s-t distribution as a response distribution. In this case, the estimation was carried out using a fully Bayesian approach with the help of Markov Chain Monte Carlo (MCMC) methods [80,81,82].

A third sensitivity analysis assessed the potential bias resulting from the telephone follow-up by study nurses on the results of the primary and secondary endpoint analyses. For both endpoints, a subgroup analysis was conducted in participants with no study nurse intervention.

#### 2.5.7. Exploratory Subgroup Analyses

Post hoc exploratory subgroup analyses were performed for the primary and secondary endpoints, considering control and intervention groups, to assess whether the treatment effects vary based on different tinnitus-relevant characteristics. The following subgroup categories were examined: age (18–49, 49–58, 58–82 years), sex (male, female), health literacy (inadequate, problematic, sufficient, excellent; see Section 2.4), tinnitus perception (tonal, atonal, tonal/atonal), hearing problems (yes, no), use of hearing aids (yes, no), sensitivity to noise (never, rarely, sometimes, usually yes, always), number of tinnitus treatments (0, 1, 2–4, ≥5), tinnitus loudness (low, medium, high), tinnitus annoyance (strong, moderate to low, none), speech comprehension problems (very strong to strong, moderate to low, none), taking medication for tinnitus (yes, no), other forms of tinnitus therapy in the last 14 days (yes, no). Furthermore, in patients of the intervention group, a subgroup analysis was conducted considering app usage (low, medium and high, defined, respectively, by the tertiles of app usage frequency: 0–20, 20–31.7, and 31.7–38 processed app units). EMM and 95% CI were calculated using a linear mixed regression model, as described above (Section 2.5.4). *p*-values for pairwise subgroup comparisons were based on undirected hypotheses and were thus two-sided.

#### 2.5.8. Analysis Software Programs

Statistical analyses were performed in R version 4.2.2 (R Core Team 2021; [66]). For the REML estimation of the mixed regression models, the R package lme4 [83] was used. For the MCMC estimation, the R package brms [84,85] was employed. For the full Bayesian estimation, four independent MCMC chains were generated with at least 10,000 iterations per chain. Convergence diagnostics were carried out graphically using the potential scale reduction factor [86], with a value of 1.01 as the upper limit for each estimated parameter. For RR calculation, the function riskratio from the package epitools [87] was used. The mixed regression models necessary for adjusting the RR for binomially distributed target variables with a logit link were estimated using the function glmer from the lme4 package [83]. Hedges’ g calculation based on the estimated unstandardized regression coefficients of the mixed models were carried out using the function esc_B from the esc package [88].

## 3. Results

### 3.1. Patients’ Demographics and Characteristics

**Figure 1:**
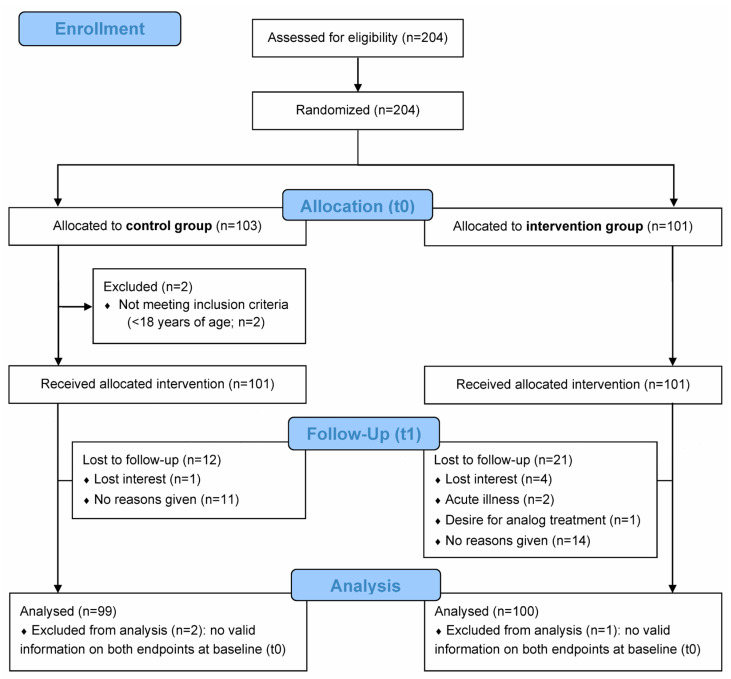
CONSORT flow diagram. Recruited patients were randomized into routine care by the ENT practitioner (control group) or routine care with additional digital counseling using the “Meine Tinnitus App” (intervention group) over a period of 10 weeks. The primary endpoint was the change in tinnitus-related distress from baseline (t0) to week 10 (endline; t1) based on the Mini-TQ-12 questionnaire score. The secondary endpoint was the change in daily burden and in coping difficulties associated with tinnitus from t0 to t1 based on the BVB-2000 questionnaire score. Analyses were conducted on the full analysis set (FAS), defined as all patients with baseline (t0) data for both primary and secondary endpoints.

A total of 204 patients with confirmed subjective tinnitus were enrolled and randomized by ENT practitioners (Figure 1). Of the 204 randomized patients, 103 were allocated to the control group and 101 to the intervention group. After excluding two patients not meeting the inclusion criteria, 101 patients each per group received the allocated intervention. Of these, 99 patients (control group) and 100 patients (intervention group) with complete baseline data were, respectively, included in the final analysis. A number of patients (12/99 [12.1%] in the control group and 21/100 [21.0%] in the intervention group) had missing endline data (Figure 1), which were accounted for using imputation methods (see Section 2.5.2).

Demographic characteristics at baseline did not differ significantly between the control and intervention groups (*p*-values > 0.05; Table 1). Age of patients in the study population ranged from 18 to 82 years, with a mean (SD) of 52.3 (12.5) years. Of the 199 analyzed participants, 50.8% were males and 49.2% females (Table 1). Most patients perceived their tinnitus as tonal (70.4%) and loud (75.9% medium to high), expressed some level of sensitivity to noise (70.3% sometimes to always), of tinnitus-associated annoyance (98.5% low to strong), and of speech comprehension problems (73.9% low to very strong). About half (50.3%) of the patients had hearing problems, although most (90.5%) did not carry a hearing aid. Up to 64.3% of participants had already received treatment for tinnitus (from 1 to ≥5 treatments) prior to start of the study, although most (87.4%) did not take medication at the time of enrollment, nor did they receive other forms of therapy (97.5%) (Table 1).

### 3.2. Change in Tinnitus-Related Distress Following 10 Weeks of Using Digital Tinnitus Counseling (Primary Endpoint)

**Figure 2:**
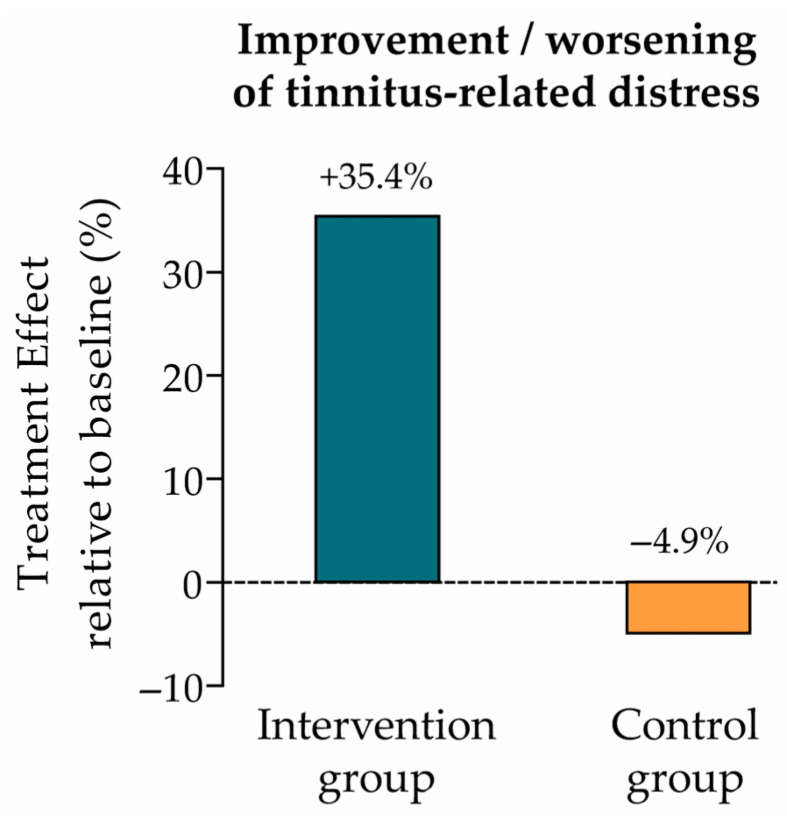
Treatment effect relative to baseline (primary endpoint: tinnitus-related distress). Improvement vs. worsening of tinnitus-related distress in the intervention and control groups, calculated as the ratio of the EMM (change from t0 to t1) to the baseline mean score (intervention group: 3.9/11.0 = +35.4%; control group: −0.6/12.3 = −4.9%; see Table 2).

The change in tinnitus-related distress between baseline (t0) and endline (t1) was evaluated using the Mini-TQ-12 questionnaire. The treatment effect from t0 to t1 was measured in each study group by calculating the estimated marginal mean (EMM) using a linear mixed regression model. EMMs (SE) in the intervention and control groups were 3.9 (0.5) and −0.6 (0.5), respectively (Table 2), which correspond to a 35.4% improvement (intervention group) and 4.9% worsening (control group) of tinnitus-related distress relative to the baseline mean score (t0) (Figure 2).

The difference in treatment effect between the intervention and control groups was statistically significant (*p* < 0.001) with an EMM [95% CI] of 4.5 [3.3–5.8] (Table 2). The practical relevance of this group comparison was calculated using standardized mean differences, which showed a large effect size (Hedges’ g = 1.1) (Table 2).

The robustness of these primary endpoint results was confirmed by several sensitivity analyses. First, the analysis was repeated without imputation of missing t1 data, i.e., in patients with a complete t0 and t1 dataset (n = 89 in the control group and n = 80 in the intervention group; Table S1). Second, the analysis was performed assuming a Student’s t-distribution instead of a normal distribution (Table S2). Third, an analysis considering the potential influence of study nurse telephone follow-up was conducted in patients without study nurse follow-up (n = 92 in the control group and n = 90 in the intervention group; Table S3). All three sensitivity analysis results (Tables S1–S3) were comparable to those of the primary endpoint results (Table 2). In the third sensitivity analysis, a pairwise comparison of the treatment difference between the larger group without study nurse follow-up and the smaller group with study nurse follow-up showed no statistically significant difference (*p* = 0.511; Table S3), indicating no apparent influence of the ‘study nurse’ factor on the primary endpoint results.

### 3.3. Responder Analysis According to the Severity Level of Tinnitus Distress

**Figure 3:**
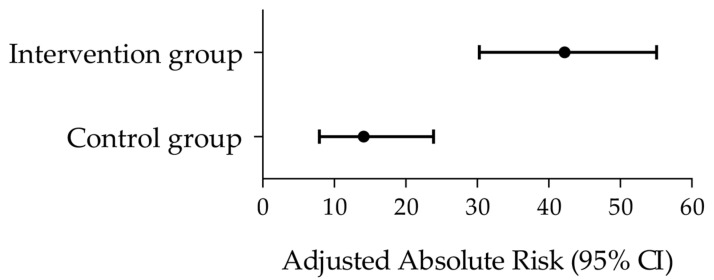
Responder analysis according to the Mini-TQ-12-based tinnitus severity classification. Forest plot depicting the adjusted absolute risks (black circles) and 95% CI (horizontal bars) in the control and intervention groups. The corresponding adjusted relative risk (95% CI) between groups was 3.0 (1.6–5.5) (see Table 3).

Practical relevance of the treatment difference between the intervention and the control groups was further evaluated with a responder analysis considering the tinnitus severity grades used in clinical routine based on the Mini-TQ-12 scores (mild, moderate, severe, extremely severe; see Section 2.5.5) [52,53]. Improvement by at least one level along this tinnitus severity classification was considered as response criterion. In the intervention group, 43.8% of patients achieved the responder criterion (improvement by up to three levels) vs. 16.9% in the control group (improvement by up to one level) (Table 3). Adjusted risks in the control and interventions groups are depicted in Figure 3. The relative risk (RR) to reach the responder criterion was 2.6 times higher in the intervention group than in the control group, and 3.0 times higher considering the adjusted RR (ARR) (Table 3).

Of note, only six of 80 (7.5%) patients in the intervention group showed a deterioration in tinnitus severity grade (down to one level along the severity scale), while 29/89 (32.6%) patients in the control group showed a deterioration in tinnitus severity (down to one or two levels).

### 3.4. Change in Daily Burden and Coping Difficulties Associated with Tinnitus, Following 10 Weeks of Using Digital Tinnitus Counseling (Secondary Endpoint)

**Figure 4:**
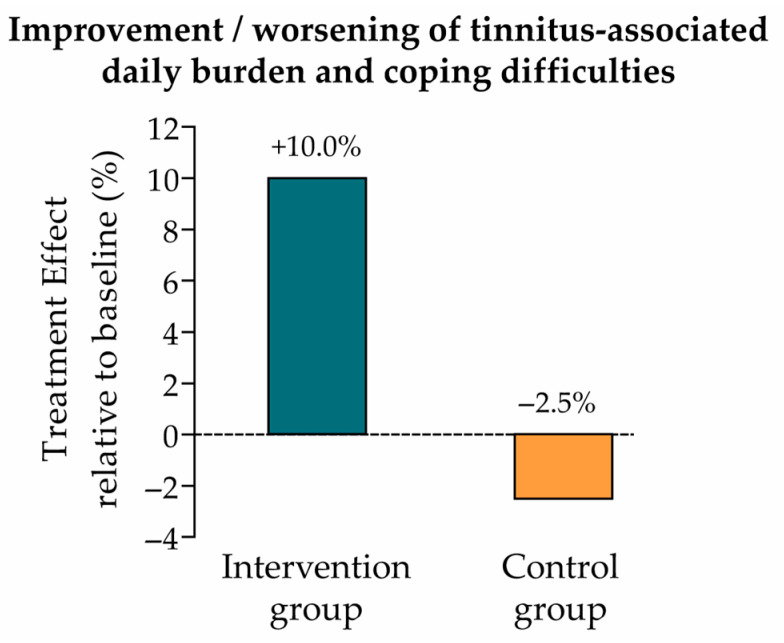
Treatment effect relative to baseline (secondary endpoint: tinnitus-associated daily burden and coping difficulties). Improvement vs. worsening of tinnitus-associated daily burden and coping difficulties in the intervention and control groups, calculated as the ratio of the EMM (change from t0 to t1) to the baseline mean score (intervention group: 0.4/4.0 = +10.0%; control group: −0.1/4.0 = −2.5%; see Table 4).

The change from baseline (t0) to endline (t1) in everyday burden and coping difficulties associated with tinnitus was evaluated using the BVB-2000 questionnaire. The treatment effect from t0 to t1 was measured by the EMM, as for the primary endpoint analysis. EMM (SE) in the intervention and control groups were 0.4 (0.1) and −0.1 (0.1), respectively (Table 4), which correspond to a 10.0% improvement (intervention group) and 2.5% worsening (control group) of tinnitus-associated daily burden and coping relative to the BVB-2000 mean score at t0 (Figure 4).

The difference in treatment effect between the intervention and control groups as assessed by the two-point measurement method of the BVB-2000 questionnaire was statistically significant (*p* < 0.001) with an EMM [95% CI] of 0.5 [0.2–0.7] (Table 4). The practical relevance of this treatment effect difference using standardized mean differences showed a medium effect size (Hedges’ g = 0.5) (Table 4).

We confirmed the secondary endpoint results (measuring the change from t0 to t1; Table 4) by applying the well-validated single-point measurement method of the BVB-2000 questionnaire, which is routinely used in medical practice [47,64]. Accordingly, this analysis considered the measurements at t1 to evaluate therapy success since t0, using the predictive mean matching imputation method. The difference in treatment effect between the intervention and control groups was again statistically significant (*p* < 0.001) with an EMM [95% CI] of 0.5 [0.3–0.7] (Table 5), alike the results of the t0 to t1 analysis (Table 4). The respective practical relevance using standardized mean differences showed a large effect size (Hedges’ g = 0.8) (Table 5).

The robustness of the secondary endpoint results (based on the single-point measurement approach; Table 5) was confirmed by several sensitivity analyses. First, the analysis was repeated without imputation, in patients with a complete t0 and t1 dataset (n = 89 in the control group and n = 80 in the intervention group; Table S4). Second, the analysis was repeated assuming a Student’s t-distribution (Table S5). Third, the analysis was conducted in patients without study nurse follow-up (n = 92 in the control group and n = 90 in the intervention group; Table S6). All three sensitivity analyses (Tables S4–S6) yielded results comparable to those of the secondary endpoint results (Table 5). In the latter sensitivity analysis, a pairwise comparison of the treatment difference between the groups without and with study nurse follow-up showed no statistically significant difference (*p* = 0.902; Table S6), indicating no apparent influence of the study nurse telephone follow-up on the secondary endpoint results.

Because of the comparability and robustness of the results obtained with the single-point measurement approach, and because of the clinical relevance of this measurement method, all further analyses in this study (responder analyses, sensitivity analyses, and subgroup analyses) based on the BVB-2000 questionnaire used this single-point-measurement approach.

### 3.5. Responder Analysis According to BVB-2000 Thresholds at 10 Weeks (t1)

Practical relevance of the treatment difference between the intervention and the control groups was further evaluated for the secondary endpoint. A responder analysis was conducted, considering the validated threshold values of the BVB-2000 questionnaire for a perceived improvement or worsening in behavior and experience (i.e., “improved” classification for BVB-2000 score > 4.4 and “worsened” classification for BVB-2000 score < 3.36; see Section 2.5.5) [47,64]. Accordingly, the response criterion was a BVB-2000 score at t1 > 4.4 (“improved”). In the intervention group, 43.8% of patients achieved the responder criterion vs. 15.7% in the control group (Table 6). The RR and ARR to reach the responder criterion were 2.8–2.9 times higher in the intervention group than in the control group (Table 6).

Of note, only 2/80 (2.5%) patients in the intervention group reported a perceived worsening in behavior and experience vs. 8/89 (9.0%) in the control group.

### 3.6. Exploratory Subgroup Analyses (Primary and Secondary Endpoints)

To investigate whether the treatment effects determined for the primary and secondary endpoints varied across patient subgroups, we conducted exploratory analyses using a broad spectrum of predefined tinnitus-relevant characteristics. Exploratory subgroup analyses were conducted on essential baseline demographics (age, sex, health literacy) and tinnitus-related parameters such as tinnitus perception, noise sensitivity, tinnitus loudness, tinnitus annoyance, hearing problems, use of hearing aids, number of tinnitus treatments, speech comprehension problems, taking medication for tinnitus, and other forms of tinnitus therapy. Mixed regression models and difference between intervention and control groups were applied for both endpoints. Pairwise comparisons between subgroups showed no statistically significant difference, neither for the primary endpoint (Table S7) nor for the secondary endpoint (Table S8) (*p*-values > 0.05).

**Figure 5:**
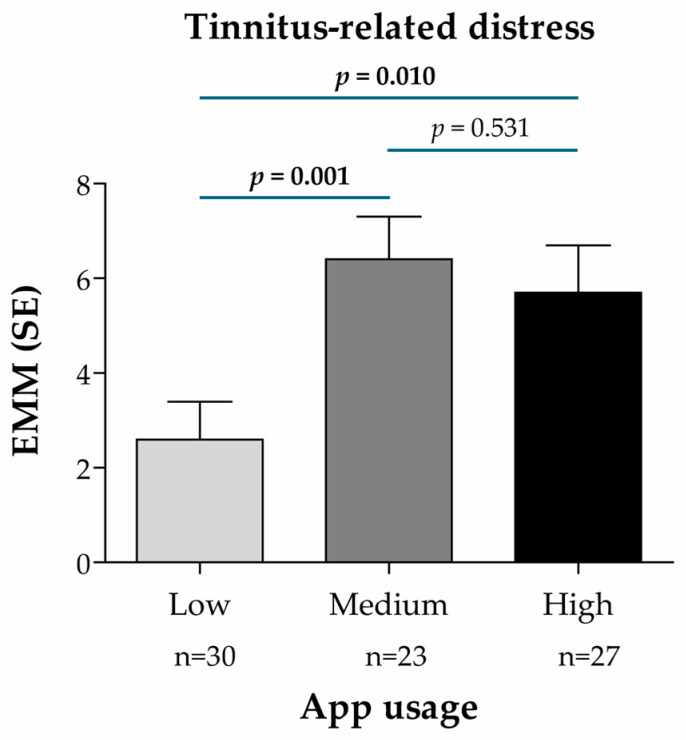
Treatment effect according to the frequency of app usage in the intervention group (n = 80), based on the Mini-TQ-12 score and predictive mean matching imputation of missing values of the target variable (primary endpoint: tinnitus-related distress). App usage subgroups were defined according to the number of app units processed and divided into terciles of app usage frequency: low (between 0 and 20 units), medium (between 20 and 31.7 units), high (between 31.7 and 38 units). EMM for the change from t0 to t1 was calculated using a linear mixed regression model with the change in the Mini-TQ-12 score as the dependent variable, baseline value and age as continuous covariates, sex as a dummy variable (0 = male, 1 = female), subgroup characteristics (app usage) as a categorical variable (reference: Low), and the ENT practice IDs as random model constants. *p*-values for pairwise subgroup comparisons are based on the non-directional hypothesis of a difference in the treatment effect between the considered subgroups (statistically significant *p*-values are highlighted in **bold**). Abbreviations: EMM, estimated marginal mean; n, number of patients per analysis group; SE, standard error.

Additionally, a subgroup analysis was conducted in patients of the intervention group (n = 80) to evaluate the treatment effect (for the primary and secondary endpoints) according to the frequency of app usage (low, medium and high, based on the number of app units processed by the participants; see Section 2.5.7). The treatment effect (EMM) for the primary endpoint was significantly higher in patients with medium (20 to 31.7 units) and high (31.7 to 38 units) usage than in those with low (0 to 20 units) usage (*p* = 0.001 and *p* = 0.010, respectively) (Figure 5), indicating a greater improvement of tinnitus-related distress together with a higher app usage.

**Figure 6:**
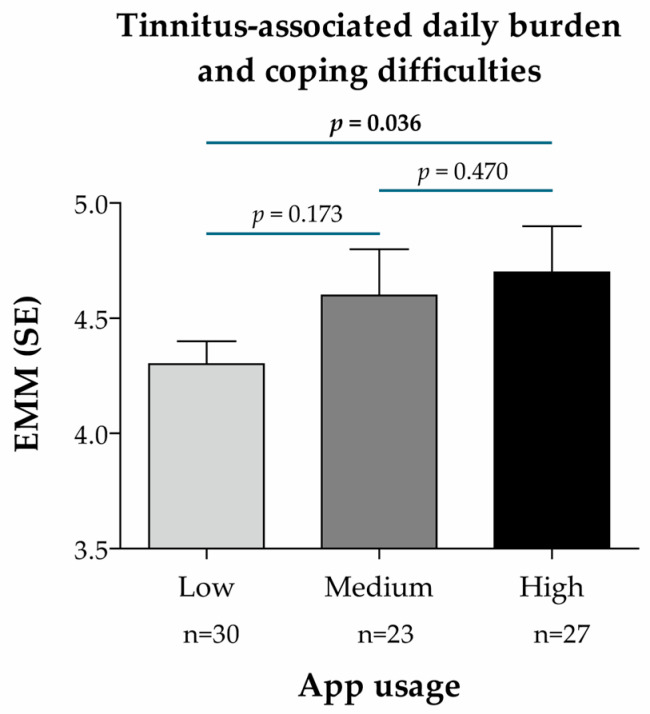
Treatment effect according to the frequency of app usage in the intervention group (n = 80), based on the BVB-2000 score (single-point measurement at t1) and predictive mean matching imputation of missing values of the target variable (secondary endpoint: tinnitus-associated daily burden and coping difficulties). App usage subgroups were defined according to the number of app units processed and divided into terciles of app usage frequency: low (between 0 and 20 units), medium (between 20 and 31.7 units), high (between 31.7 and 38 units). EMM was calculated using a linear mixed regression model with the change in the BVB-2000 score at t1 as the dependent variable, age as a continuous covariate, sex as a dummy variable (0 = male, 1 = female), subgroup characteristics (app usage) as a categorical variable (reference: Low), and the ENT practice IDs as random model constants. *p*-values for pairwise subgroup comparisons are based on the non-directional hypothesis of a difference in the treatment effect between the considered subgroups (statistically significant *p*-values are highlighted in **bold**). Abbreviations: EMM, estimated marginal mean; n, number of patients per analysis group; SE, standard error.

For the secondary endpoint, the treatment effect (EMM) was slightly higher in patients with medium and high usage than in those with low usage, the difference being statistically significant between the low and high usage groups (*p* = 0.036; Figure 6). This indicates a stronger reduction in tinnitus-associated daily burden and improved coping in patients with a high vs. low app usage.

## 4. Discussion

This two-arm randomized open-controlled multicenter study evaluated the effectiveness of tinnitus counseling provided by “Meine Tinnitus App” (MTA) as guideline-compliant digital therapy for patients with confirmed chronic subjective tinnitus.

We demonstrated a significant therapy effect, reflected in reduced tinnitus-related distress as measured by the Mini-TQ-12 questionnaire, and in decreased daily burden and coping difficulties associated with tinnitus as measured by the BVB-2000 questionnaire, in patients of the intervention group vs. the control group after 10 weeks of intervention (EMM *p* < 0.001 for both endpoints).

The benefit of app-based counseling in the intervention group was confirmed by practical relevance analyses demonstrating a strong impact of the app in reducing tinnitus-related distress (Hedges’ g of 1.1) and a moderate to strong impact in alleviating daily burden and coping difficulties (Hedges’ g of 0.5 to 0.8, depending on the measurement method). Responder analyses based on validated thresholds employed in routine medical practice and described in the literature [47,52,53,64] further confirmed practical relevance (RR and ARR between 2.6 and 3.0).

Treatment effects for both endpoints were confirmed by sensitivity analyses demonstrating no apparent data distortions due to the imputation approach, data distribution assumptions, or telephone follow-up by study nurses. As to the BVB-2000-based analyses (secondary endpoint), comparable results were obtained using the single-point measurement approach that is commonly used in clinical practice [47,64].

The clinical value of the app-based treatment was further supported by the demonstration of a significant improvement in treatment effect in patients with a higher app usage vs. a low usage (*p*-values between 0.036 and 0.001). The higher benefit of app-based treatment with greater app usage is notable, as it was observed for both the primary and secondary endpoints (tinnitus-related distress and daily burden/coping difficulties associated with tinnitus). This is important as it highlights digital adherence as a therapeutic factor. These results are in line with those of Schlee et al. showing an improvement in tinnitus-related distress in patients with greater app usage [43]. Although differences in the way various patients adhere to app usage were expected, the observation that some patients engaged less in the proposed digital counseling should drive us to identify possible approaches to encourage patients’ engagement. This may be achieved by implementing more motivational components or notifications, although using more prompts might be counterproductive by raising the patient’s awareness of their tinnitus. This risk is however limited, as reported in a study suggesting no negative impact of repeated prompts on long-term tinnitus-related distress [89].

Our study included 33 (16.6%) dropouts, most of which (n = 25) with no given reasons, while few discontinuations were due to a loss of interest (n = 5), acute illness (n = 2), or a preference for analog treatment (n = 1). Apparent reasons for dropout did not involve a smartphone-related technical issue, which speaks for the reliability and ease of use of MTA. This is important, as some researchers underlined smartphone-related technical issues as a major reason for study dropout and a possible source of bias in their study [43]. It is worth mentioning that the impact of patient dropouts between t0 and t1 in our study was corrected by the implementation of validated imputation procedures, with no apparent bias, as indicated by our sensitivity analysis results. Altogether, the app demonstrated good user acceptability, with a dropout rate (16.6%) comparable to or better than several other studies conducted within the DiGA framework, in which dropout rates at the primary endpoint survey ranged from 6.7% to 30.0%, and at the last follow-up time point from 5.9% to 61.3% [90]. Despite the good user acceptability noted in our study, one should not underestimate the implications of digital literacy and access disparities, particularly among older or technologically inexperienced populations. Such barriers might reduce the effectiveness of digital intervention in such patients. Addressing these challenges is essential to ensure equitable benefit across diverse tinnitus patient populations. The accessibility features and individualized pacing of MTA aim to support engagement across heterogeneous patient populations.

Overall, our results support the observations of other randomized controlled studies demonstrating the positive impact of digital health intervention using counseling and/or CBT components, in managing symptoms of chronic subjective tinnitus [39,40,41,42,43,44,45,91].

Exploratory subgroup analyses comparing demographic categories (age class, sex) and tinnitus-relevant characteristics (including tinnitus perception, loudness or annoyance, sensitivity to noise, hearing problems) showed no statistically significant differences between subgroups. Although some of these subgroup analyses were inconclusive due to small group sizes, these observations suggest that the treatment effect of MTA is not influenced by these essential attributes and thus that the app is applicable to all adult patients with confirmed chronic subjective tinnitus. Further studies with larger subgroup sizes should confirm this assumption. A multicenter study with a larger number of participants per center would also allow for the evaluation of between-center variability and the comparison of the impact of MTA between centers. This is a critical evaluation component, given the reported disparity in tinnitus care and counseling generally provided by ENT practices [5,6,31,32].

Our study’s strengths include the analysis of a large cohort of chronic subjective tinnitus patients (n = 199), particularly in comparison to existing randomized studies [39,40,41,42,43]. The randomized design with equally sized groups further enhances the robustness of our findings. Our extensive and complementary analyses (responder, sensitivity, and subgroup analyses), considering multiple potential confounders, all provided concordant results, strengthening evidence for the treatment effects (reduction in tinnitus-related distress and in daily burden and coping difficulties associated with tinnitus) observed in the intervention group. The low dropout rate discussed above also strengthens the value of our results. Additionally, the multicenter nature of the study adds value by better reflecting real-world conditions, notably the tinnitus management landscape in German ENT practices.

Our study includes a few limitations. The first one is the absence of blinding, which was imposed by its design (i.e., with or without app usage), the participants being fully aware of their respective treatment allocation. This non-blinding design might explain the worsening in tinnitus-related distress, daily burden and coping difficulties perceived by the control group between t0 and t1. This apparent worsening effect in the control group might be attributed, at least in part, to the patients’ awareness of missing a treatment opportunity. Of note, to rectify unfair treatment options between groups, patients of the control group were offered free access to digital counseling using MTA after completion of the study. On the other hand, we cannot exclude that the non-blinding design might contribute to a placebo or expectancy effect in patients of the intervention group, which might explain part of the improvement in treatment effect observed in this group. A second limitation of our study is the exclusion of patients with anxiety, depression and other psychiatric conditions. Given that these comorbidities are common in tinnitus patients, our data do not allow generalizability to this category of patients. Future studies should include patients with acute or chronic mental illness. A third limitation of our study is the lack of availability of audiology data that would have allowed us a more thorough evaluation of patients’ hearing ability. This flaw should be corrected in future studies. Finally, the absence of standardized tinnitus care (defined as standard of care in our study) among the 33 included ENT centers, notably in terms of tinnitus counseling content and frequency, raises the question of its potential impact on our study results. This impact is however expected to be low, given that standard of care was applied to both the intervention and control groups, and that our study evaluated the effect of app-based therapy in addition to standard care. Nevertheless, the question of standard care variability could be addressed by performing between-center comparisons in a study with larger group sizes, as already mentioned. Alternatively, a waitlist-controlled randomized trial, with patients of the control group eventually receiving app-based intervention in a subsequent study phase, could address this question, and thus correct for standard care variability between centers.

Finally, besides larger cohort studies and waitlist-controlled trials, future studies should also include a long-term follow-up (i.e., beyond the 10-week period considered in this study), to evaluate the persistence of the beneficial impact of app-based counseling and the potential need of treatment refreshment. Longitudinal studies including multiple measurement time points over a long period of time would surely provide valuable information on treatment effects over time and the long-term benefit of MTA for the treatment of chronic subjective tinnitus.

## 5. Conclusions

This study demonstrated the positive therapeutic effects of “Meine Tinnitus App” in reducing tinnitus-related distress and the daily burden and coping difficulties associated with tinnitus for patients with chronic subjective tinnitus. Hence, “Meine Tinnitus App” is an effective digital tinnitus counseling tool, assisting healthcare providers and helping patients manage their tinnitus and improve their quality of life.

## Figures and Tables

**Table 1:** Patients’ demographics and characteristics at baseline.

Characteristics	Total Population (FAS) (N = 199)	Control Group (N = 99)	Intervention Group (N = 100)	*p*-Value
Age in years, mean (SD)	52.3 (12.5)	53.4 (12.0)	51.2 (13.0)	0.210 ^1^
Sex, N (%)				0.178 ^2^
Male	101 (50.8)	45 (45.5)	56 (56.0)	
Female	98 (49.2)	54 (54.5)	44 (44.0)	
Mini-TQ-12 score, mean (SD)	11.7 (5.6)	12.3 (5.6)	11.0 (5.4)	0.104 ^1^
BVB-2000 score, mean (SD)	4.0 (0.7)	4.0 (0.8)	4.0 (0.5)	0.436 ^1^
Health literacy, N (%)				0.585 ^2^
Inadequate	26 (13.1)	10 (0.1)	16 (16.0)	
Problematic	66 (33.2)	36 (36.4)	30 (30.0)	
Sufficient	75 (37.7)	37 (37.4)	38 (38.0)	
Excellent	32 (16.1)	16 (16.2)	16 (16.0)	
Tinnitus perception, N (%)				0.977 ^2^
Tonal	140 (70.4)	69 (69.7)	71 (71.0)	
Atonal	43 (21.6)	22 (22.2)	21 (21.0)	
Tonal/Atonal	16 (8.0)	8 (8.1)	8 (8.0)	
Hearing problems, N (%)				0.944 ^2^
Yes	100 (50.3)	49 (49.5)	51 (51.0)	
No	99 (49.7)	50 (50.5)	49 (49.0)	
Use of hearing aids, N (%)				1.000 ^2^
Yes	19 (9.5)	9 (9.1)	10 (10.0)	
No	180 (90.5)	90 (90.9)	90 (90.0)	
Sensitivity to noise, N (%)				0.714 ^2^
Never	16 (8.0)	10 (10.1)	6 (6.0)	
Rarely	43 (21.6)	23 (23.2)	20 (20.0)	
Sometimes	81 (40.7)	37 (37.4)	44 (44.0)	
Usually yes	45 (22.6)	23 (23.2)	22 (22.0)	
Always	14 (7.0)	6 (6.1)	8 (8.0)	
Number of tinnitus treatments, N (%)				0.342 ^3^
0	67 (33.7)	34 (34.3)	33 (33.0)	
1	51 (25.6)	24 (24.2)	27 (27.0)	
2–4	56 (28.1)	24 (24.2)	32 (32.0)	
≥5	21 (10.6)	14 (14.1)	7 (7.0)	
Not specified	4 (2.0)	3 (3.0)	1 (1.0)	
Tinnitus loudness, N (%)				0.175 ^2^
Low	48 (24.1)	20 (20.2)	28 (28.0)	
Medium	90 (45.2)	43 (43.4)	47 (47.0)	
High	61 (30.7)	36 (36.4)	25 (25.0)	
Tinnitus annoyance, N (%)				0.143 ^3^
Strong	73 (36.7)	40 (40.4)	33 (33.0)	
Moderate to low	123 (61.8)	56 (56.6)	67 (67.0)	
None	1 (0.5)	1 (1.0)	0 (0.0)	
Not specified	2 (1.0)	2 (2.0)	0 (0.0)	
Language comprehension problems, N (%)				0.503 ^3^
Very strong to strong	45 (22.6)	26 (26.3)	19 (19.0)	
Moderate to low	102 (51.3)	49 (49.5)	53 (53.0)	
None	51 (25.6)	24 (24.2)	27 (27.0)	
Not specified	1 (0.5)	0 (0.0)	1 (1.0)	
Medication intake against tinnitus, N (%) ^4^				1.000 ^2^
Yes	25 (12.6)	12 (12.1)	13 (13.0)	
No	174 (87.4)	87 (87.9)	87 (87.0)	
Other forms of therapy, N (%) ^4^				1.000 ^3^
Yes	5 (2.5)	2 (2.0)	3 (3.0)	
No	194 (97.5)	97 (98.0)	97 (97.0)	

^1^ *t*-Test. ^2^ Chi-Square (χ^2^) Test. ^3^ Fisher’s Exact Test. ^4^ At the time of study inclusion (t0). Abbreviations: N, number of patients; SD, standard deviation.

**Table 2:** Primary endpoint analysis (Mini-TQ-12 score) with jump-to-reference imputation of missing t1 data (FAS); primary endpoint results without imputation are shown in Table S1.

Treatment Group	Baseline t0		Endline t1	Change from t0 to t1	Treatment Difference from t0 to t1 (Control vs. Intervention)
	FAS n Mean (SD)	FAS with t1 Data n Mean (SD)	FAS with t1 Data n Mean (SD)	FAS n Mean Difference (SE) EMM ^1^ (SE)	
Control group (N = 99)	99	89	89	99	**EMM [95% CI]** 4.5 [3.3–5.8] *p* < 0.001 ^2^ **Hedges’ g [95% CI]** 1.1 [0.8–1.4]
	12.3	12.0	12.7	−0.6 (0.3)	
	(5.6)	(5.6)	(6.1)	−0.6 (0.5)	
Intervention group (N = 100)	100	80	80	100	
	11.0 (5.4)	11.1 (5.7)	6.9 (5.1)	2.9 (0.5) 3.9 (0.5)	

^1^ Calculated using a linear mixed regression model with the change in the Mini-TQ-12 score as the dependent variable, intervention as a dummy variable (0 = control group, 1 = intervention group), baseline value and age as continuous covariates, sex as a dummy variable (0 = male, 1 = female) and the ENT practice IDs as random model constants. ^2^ *p*-value based on the directed hypothesis of a greater improvement in the intervention group. Abbreviations: CI, confidence interval; EMM, estimated marginal mean; ENT, Ear, Nose and Throat medical specialty; FAS, full analysis set; N, number of patients included in the study; n, number of patients included in the analysis; SD, standard deviation; SE, standard error; t0, baseline (study start); t1, endline (10 weeks after study start).

**Table 3:** Responder analysis according to the Mini-TQ-12-based tinnitus severity classification, with jump-to-reference imputation of missing values of the response variable (FAS).

Treatment Group	Responder ^1^ n (%)	RR [95% CI]	ARR ^2^ [95% CI]
Control group (N = 89)	15 (16.9)	2.6 [1.5–4.4]	3.0 [1.6–5.5]
Intervention group (N = 80)	35 (43.8)		

^1^ Response criterion: improvement by at least one level within the Mini-TQ-12 tinnitus severity classification (mild, moderate, severe, extremely severe). ^2^ Calculated using a mixed regression model for binomially distributed outcome variables with logit link function, intervention as dummy variable (0 = control group, 1 = intervention group), baseline value and age as continuous covariates, sex as dummy variable (0 = male, 1 = female) and the ENT practice IDs as random model constants. ARR was calculated by back-transforming the estimated marginal risks using the natural logarithm. Abbreviations: ARR, adjusted relative risk; CI, confidence interval; ENT, Ear, Nose and Throat medical specialty; FAS, full analysis set; N, number of study patients with complete data; n, number of patients meeting the response criterion; RR, relative risk.

**Table 4:** Secondary endpoint analysis (BVB-2000 score) with jump-to-reference imputation of missing t1 data (FAS).

Treatment Group	Baseline t0		Endline t1	Change from t0 to t1	Treatment Difference from t0 to t1 (Control vs. Intervention)
	FAS n Mean (SD)	FAS with t1 Data n Mean (SD)	FAS with t1 Data n Mean (SD)	FAS n Mean Difference (SE) EMM ^1^ (SE)	
Control group (N = 99)	99	89	89	99	**EMM [95% CI]** 0.5 [0.2–0.7] *p* < 0.001 ^2^ **Hedges’ g [95% CI]** 0.5 [0.3–0.8]
	4.0	4.0	4.0	0.0 (0.1)	
	(0.8)	(0.8)	(0.7)	−0.1 (0.1)	
Intervention group (N = 100)	100	80	80	100	
	4.0 (0.5)	4.0 (0.6)	4.5 (0.7)	0.3 (0.1) 0.4 (0.1)	

^1^ Calculated using a linear mixed regression model with the change in the BVB-2000 score as the dependent variable, intervention as a dummy variable (0 = control group, 1 = intervention group), baseline value and age as continuous covariates, sex as a dummy variable (0 = male, 1 = female) and the ENT practice IDs as random model constants. ^2^ *p*-value based on the directed hypothesis of a greater improvement in the intervention group. Abbreviations: CI, confidence interval; EMM, estimated marginal mean; ENT, Ear, Nose and Throat medical specialty; FAS, full analysis set; N, number of patients included in the study; n, number of patients included in the analysis; SD, standard deviation; SE, standard error; t0, baseline (study start); t1, endline (10 weeks after study start).

**Table 5:** Secondary endpoint analysis (BVB-2000) as single-point measurement with predictive mean matching imputation of missing t1 data (FAS); secondary endpoint results without imputation are shown in Table S4.

Treatment Group	t1 Measurement	Estimated Value at t1	Treatment Difference at t1 (Control vs. Intervention)
	FAS with t1 Data n Mean (SD)	FAS n Mean Difference (SE) EMM ^1^ (SE)	
Control group (N = 99)	89	99	**EMM [95% CI]** 0.5 [0.3–0.7] *p* < 0.001 ^2^ **Hedges’ g [95% CI]** 0.8 [0.5–1.0]
	4.0	4.0 (0.1)	
	(0.7)	4.0 (0.1)	
Intervention group (N = 100)	80	100	
	4.5 (0.7)	4.5 (0.1) 4.5 (0.1)	

^1^ Calculated using a linear mixed regression model with the change in the BVB-2000 score at t1 as the dependent variable, intervention as a dummy variable (0 = control group, 1 = intervention group), age as continuous covariate, sex as a dummy variable (0 = male, 1 = female) and the ENT practice IDs as random model constants. ^2^ *p*-value based on the directed hypothesis of a greater improvement in the intervention group. Abbreviations: CI, confidence interval; EMM, estimated marginal mean; ENT, Ear, Nose and Throat medical specialty; FAS, full analysis set; N, number of patients included in the study; n, number of patients included in the analysis; SD, standard deviation; SE, standard error; t1, endline (10 weeks after study start).

**Table 6:** Responder analysis according to the BVB-2000-based scoring categories, with single-point measurement and predictive mean matching imputation of missing values of the response variable (FAS).

Treatment Group	Responder ^1^ n (%)	RR [95% CI]	ARR ^2^ [95% CI]
Control group (N = 89)	14 (15.7)	2.8 [1.6–4.8]	2.9 [1.7–4.9]
Intervention group (N = 80)	35 (43.8)		

^1^ Response criterion: BVB-2000 score > 4.4 (“improved”) at t1. ^2^ Calculated using a mixed regression model for binomially distributed outcome variables with logit link function, intervention as dummy variable (0 = control group, 1 = intervention group), baseline value and age as continuous covariates, sex as dummy variable (0 = male, 1 = female) and the ENT practice IDs as random model constants. ARR was calculated by back-transforming the estimated marginal risks using the natural logarithm. Abbreviations: ARR, adjusted relative risk; CI, confidence interval; ENT, Ear, Nose and Throat medical specialty; FAS, full analysis set; N, number of study patients with complete data; n, number of patients meeting the response criterion; RR, relative risk.

## Data Availability

The original contributions presented in this study are included in the article/Supplementary Material. Further inquiries can be directed to the corresponding author.

## Supplementary Materials

The following supporting information can be downloaded at: https://www.mdpi.com/article/10.3390/audiolres15060173/s1, Table S1: Primary endpoint analysis (Mini-TQ-12 score) without imputation (FAS); Table S2: Primary endpoint analysis (Mini-TQ-12 score) with jump-to-reference imputation of missing t1 data and assuming Student’s t-distribution (FAS); Table S3: Primary endpoint analysis (Mini-TQ-12 score) with jump-to-reference imputation of missing t1 data, comparing subgroups of patients with and without study nurse telephone follow-up; Table S4: Secondary endpoint analysis (BVB-2000) as single-point measurement at t1 without imputation of missing values (FAS); Table S5: Secondary endpoint analysis (BVB-2000) as single-point measurement at t1 with predictive mean matching imputation of missing values and assuming Student’s t-distribution (FAS); Table S6: Secondary endpoint analysis (BVB-2000 score) as single-point measurement at t1 with predictive mean matching imputation of missing values, comparing subgroups of patients with and without study nurse telephone follow-up; Table S7: Primary endpoint analysis (Mini-TQ-12 score) with jump-to-reference imputation of missing t1 data, comparing diverse subgroups of patients; Table S8: Secondary endpoint analysis (BVB-2000 score) as single-point measurement at t1 with predictive mean matching imputation of missing values, comparing diverse subgroups of patients.

## Abbreviations

The following abbreviations are used in this manuscript:

ARR	Adjusted relative risk
BVB-2000	Bochumer Veränderungsbogen-2000 (standardized questionnaire to quantify subjectively perceived alterations under treatment, which can be used to measure changes in well-being and behavior (such as daily burden and coping difficulties) in the course of therapy)
CBT	Cognitive behavioral therapy
CI	Confidence interval
EMM	Estimated marginal mean
ENT	Ear, Nose and Throat (medical specialty)
FAS	Full analysis set
Mini-TQ-12	Short version of the tinnitus questionnaire (TQ) with 12 questions
MTA	Meine Tinnitus App (registered name; German for “My Tinnitus App”)
RR	Relative risk
SAP	Statistical analysis plan
SD	Standard deviation
SE	Standard error
t0	Baseline (study inclusion and first primary and secondary endpoint measurements)
t1	Endline (10 weeks after t0; second primary and secondary endpoint measurements)
