# Supplementary material for: Digital Tinnitus Counseling in Clinical Practice: A Multicenter Randomized Controlled Trial
Source: Audiol Res. 2025 Dec 9;15(6):173. doi: 10.3390/audiolres15060173 (PMC12729911; doi:10.3390/audiolres15060173)
Supplement: Supplementary file 1 [file audiolres-15-00173-s001.zip › audiolres-3930114-supplementary.pdf]

Supplementary Tables

Table S1. Primary endpoint analysis (Mini-TQ-12 score) without imputation (FAS); primary endpoint results with imputation of missing t1 data are shown in Table 2.

**Treatment group**	**Baseline t0**<br>**FAS**<br>**n**<br>**Mean**<br>**(SD)**	 **FAS with t1 data**<br>**n**<br>**Mean**<br>**(SD)**	**Endline t1**<br>**FAS with t1 data**<br>**n**<br>**Mean**<br>**(SD)**	**Change from t0 to t1**<br>**FAS**<br>**n**<br>**Mean difference (SE)**<br>**EMM [1] (SE)**	**Treatment difference from t0 to t1 (control vs. intervention)**
Control group<br>(N = 99)	99<br>12.3<br>(5.6)	89<br>12.0<br>(5.6)	89<br>12.7<br>(6.1)	89<br>-0.7 (0.4)<br>-0.5 (0.5)	**EMM [95% CI]**<br>5.1 [3.8 – 6.3]<br>$p < 0.001$ [2]
Intervention group<br>(N = 100)	100<br>11.0<br>(5.4)	80<br>11.1<br>(5.7)	80<br>6.9<br>(5.1)	80<br>4.2 (0.6)<br>4.5 (0.5)	**Hedges' g [95% CI]**<br>1.2 [0.9 – 1.5]

[1] Calculated using a linear mixed regression model with the change in the Mini-TQ-12 score as the dependent variable, intervention as a dummy variable (0 = control group, 1 = intervention group), baseline value and age as continuous covariates, sex as a dummy variable (0 = male, 1 = female) and the ENT practice IDs as random model constants. Treatment effect relative to baseline, calculated as the ratio of the EMM of the change from t0 to t1 to the mean score at t0, was +40.9% in the intervention group (improvement) and -4.1% in the control group (worsening) (see Figure 2 for the respective results with imputation of missing t1 data). [2] *p*-value based on the directed hypothesis of a greater improvement in the intervention group. Abbreviations: CI, confidence interval; EMM, estimated marginal mean; ENT, Ear, Nose and Throat medical specialty; FAS, full analysis set; N, number of patients included in the study; n, number of patients included in the analysis; SD, standard deviation; SE, standard error; t0, baseline (study start); t1, endline (10 weeks after study start).

Table S2. Primary endpoint analysis (Mini-TQ-12 score) with jump-to-reference imputation of missing t1 data and assuming Student's t-distribution (FAS).

Treatment group	Baseline t0<br>FAS	 FAS with t1 data	Endline t1<br>FAS with t1 data	Change from t0 to t1<br>FAS	Treatment difference from t0 to t1 (control vs. intervention)
	n<br>Mean<br>(SD)	n<br>Mean<br>(SD)	n<br>Mean<br>(SD)	n<br>Mean difference (SE)<br>EMM [1] (SE)	
Control group<br>(N = 99)	99<br>12.3<br>(5.6)	89<br>12.0<br>(5.6)	89<br>12.7<br>(6.1)	99<br>-0.6 (0.3)<br>-0.4 (0.7)	**EMM [95% CI]**<br>3.7 [2.1 – 5.4]<br>$p > 0.999$ [2]<br>**Hedges' g [95% CI]**<br>0.9 [0.6 – 1.2]
Intervention group<br>(N = 100)	100<br>11.0<br>(5.4)	80<br>11.1<br>(5.7)	80<br>6.9<br>(5.1)	100<br>2.9 (0.5)<br>3.3 (0.9)	

[1] Calculated using hierarchical regression model with Student's t-distribution as the target variable distribution. The dependent variable is the change in the Mini-TQ-12 score, intervention a dummy variable (0 = control group, 1 = intervention group), baseline value and age continuous covariates, sex a dummy variable (0 = male, 1 = female) and the ENT practice IDs random model constants. Treatment effect relative to baseline, calculated as the ratio of the EMM of the change from t0 to t1 to the mean score at t0, was +30.0% in the intervention group (improvement) and -3.2% in the control group (worsening). [2] *p*-value is based on posterior probabilities for superiority of the intervention group over the control group (a high *p*-value is associated with a significant treatment effect). Abbreviations: CI, confidence interval; EMM, estimated marginal mean; ENT, Ear, Nose and Throat medical specialty; FAS, full analysis set; N, number of patients included in the study; n, number of patients included in the analysis; SD, standard deviation; SE, standard error; t0, baseline (study start); t1, endline (10 weeks after study start).

Table S3. Primary endpoint analysis (Mini-TQ-12 score) with jump-to-reference imputation of missing t1 data, comparing subgroups of patients with and without study nurse telephone follow-up.

Study nurse follow-up	Change from t0 to t1				Treatment difference from t0 to t1 (control vs. intervention) EMM [1] [95% CI]	Pairwise subgroup comparison of treatment difference *p*-value [2]
	Control group		Intervention group			
	n	EMM [1] (SE)	n	EMM [1] (SE)		
No (N = 182)	92	-0.6 (0.5)	90	4.0 (0.6)	4.6 [3.3 – 5.9]	0.511
Yes (N = 17)	7	0.2 (1.6)	10	3.4 (1.4)	3.1 [-1.1 – 7.3]	
Total (N = 199)	99	-0.6 (0.5)	100	3.9 (0.5)	4.5 [3.3 – 5.8]	

[1] Calculated using a linear mixed regression model with the change in the Mini-TQ-12 score as the dependent variable, intervention as a dummy variable (0 = control group, 1 = intervention group), baseline value and age as continuous covariates, sex as a dummy variable (0 = male, 1 = female), subgroup characteristics (study nurse follow-up) as a categorical variable (No/Yes; reference: No), the interaction between the intervention variable and the subgroup characteristics as well as the ENT practice IDs as random model constants. [2] *p*-value based on the non-directional hypothesis of a difference in the intervention variable between the considered subgroups and calculated based on the corresponding regression coefficient of the interaction term. Abbreviations: CI, confidence interval; EMM, estimated marginal mean; ENT, Ear, Nose and Throat medical specialty; FAS, full analysis set; N, number of patients per subgroup or in total; n, number of patients per analysis group; SE, standard error; t0, baseline (study start); t1, endline (10 weeks after study start).

Table S4. Secondary endpoint analysis (BVB-2000) as single-point measurement at t1 without imputation of missing values (FAS); secondary endpoint results with imputation of missing t1 data are shown in Table 5.

Treatment group	t1 measurement FAS with t1 data<br>n<br>Mean<br>(SD)	Estimated value at t1 FAS<br>n<br>Mean difference (SE)<br>EMM [1] (SE)	Treatment difference at t1 (control vs. intervention)
Control group (N = 99)	89<br>4.0<br>(0.7)	99<br>4.0 (0.1)<br>4.0 (0.1)	**EMM [95% CI]**<br>0.5 [0.3 – 0.8]<br>$p < 0.001$ [2]<br>**Hedges' g [95% CI]**<br>0.8 [0.5 – 1.1]
Intervention group (N = 100)	80<br>4.5<br>(0.7)	100<br>4.5 (0.1)<br>4.5 (0.1)	

[1] Calculated using a linear mixed regression model with the change in the BVB-2000 score at t1 as the dependent variable, intervention as a dummy variable (0 = control group, 1 = intervention group), age as continuous covariate, sex as a dummy variable (0 = male, 1 = female) and the ENT practice IDs as random model constants. [2] p-value based on the directed hypothesis of a greater improvement in the intervention group. Abbreviations: CI, confidence interval; EMM, estimated marginal mean; ENT, Ear, Nose and Throat medical specialty; FAS, full analysis set; N, number of patients included in the study; n, number of patients included in the analysis; SD, standard deviation; SE, standard error; t1, endline (10 weeks after study start).

Table S5. Secondary endpoint analysis (BVB-2000) as single-point measurement at t1 with predictive mean matching imputation of missing values and assuming Student's t-distribution (FAS).

Treatment group	t1 measurement<br>FAS with t1 data	Estimated value at t1<br>FAS	Treatment difference at t1 (control vs. intervention)
	n<br>Mean<br>(SD)	n<br>Mean difference (SE)<br>EMM [1] (SE)	
Control group<br>(N = 99)	89<br>4.0<br>(0.7)	99<br>4.0 (0.1)<br>4.0 (0.1)	**EMM [95% CI]**<br>0.4 [0.3 – 0.6]<br>$p > 0.999$ [2]
Intervention group<br>(N = 100)	80<br>4.5<br>(0.7)	100<br>4.5 (0.1)<br>4.5 (0.1)	**Hedges' g [95% CI]**<br>0.6 [0.3 – 0.9]

[1] Calculated using a hierarchical regression model with Student's t-distribution as the target variable distribution. The dependent variable is the change in the BVB-2000 score at t1, intervention a dummy variable (0 = control group, 1 = intervention group), age a continuous covariate, sex a dummy variable (0 = male, 1 = female) and the ENT practice IDs random model constants. [2] *p*-value is based on posterior probabilities for superiority of the intervention group over the control group (a high *p*-value is associated with a significant treatment effect). Abbreviations: CI, confidence interval; EMM, estimated marginal mean; ENT, Ear, Nose and Throat medical specialty; FAS, full analysis set; N, number of patients included in the study; n, number of patients included in the analysis; SD, standard deviation; SE, standard error; t1, endline (10 weeks after study start).

Table S6. Secondary endpoint analysis (BVB-2000 score) as single-point measurement at t1 with predictive mean matching imputation of missing values, comparing subgroups of patients with and without study nurse telephone follow-up.

Study nurse follow-up	Single-point measurement at t1 Control group		Intervention group		Treatment difference at t1 (control vs. intervention) EMM [1] [95% CI]	Pairwise subgroup comparison of treatment difference *p*-value [2]
	n	EMM [1] (SE)	n	EMM [1] (SE)		
No (N = 182)	92	4.0 (0.1)	90	4.5 (0.1)	0.5 [0.3 – 0.8]	0.902
Yes (N = 17)	7	3.7 (0.3)	10	4.2 (0.2)	0.5 [-0.2 – 1.2]	
Total (N = 199)	99	4.0 (0.1)	100	4.5 (0.1)	0.5 [0.3 – 0.7]	

[1] Calculated using a linear mixed regression model with the change in the BVB-2000 score at t1 as the dependent variable, intervention as a dummy variable (0 = control group, 1 = intervention group), age as a continuous covariate, sex as a dummy variable (0 = male, 1 = female), subgroup characteristics (study nurse follow-up) as a categorical variable (No/Yes; reference: No), the interaction between the intervention variable and the subgroup characteristics as well as the ENT practice IDs as random model constants. [2] *p*-value based on the non-directional hypothesis of a difference in the intervention variable between the considered subgroups and calculated based on the corresponding regression coefficient of the interaction term. Abbreviations: CI, confidence interval; EMM, estimated marginal mean; ENT, Ear, Nose and Throat medical specialty; FAS, full analysis set; N, number of patients per subgroup or in total; n, number of patients per analysis group; SE, standard error; t1, endline (10 weeks after study start).

Table S7. Primary endpoint analysis (Mini-TQ-12 score) with jump-to-reference imputation of missing t1 data, comparing diverse subgroups of patients.

**Subgroups**		**Change from t0 to t1**				**Treatment difference from t0 to t1 (control vs. intervention) EMM [1] [95% CI]**	**Pairwise subgroup comparison of treatment difference *p*-value [2]**			
		**Control group**		**Intervention group**						
		**n**	**EMM [1] (SE)**	**n**	**EMM [1] (SE)**					
	Total (N = 199)	99	-0.6 (0.5)	100	3.9 (0.5)	4.5 [3.3 – 5.8]				
**Age (years)**							**49–58**	**58-82**		
	**18–49** (N=69)	27	0.1 (0.9)	42	3.9 (0.7)	3.8 [1.7 – 5.9]	0.278	0.725		
	**49–58** (N=66)	37	-0.6 (0.8)	29	5.0 (0.9)	5.5 [3.3 – 7.8]	-	0.478		
	**58–82** (N=64)	35	-1.2 (0.8)	29	3.2 (0.9)	4.4 [2.1 – 6.7]	-	-		
**Sex**							**Female**			
	**Male** (N=101)	45	-0.6 (0.7)	56	3.4 (0.7)	4.1 [2.4 – 5.8]	0.483			
	**Female** (N=98)	54	-0.5 (0.7)	44	4.5 (0.7)	5.0 [3.1 – 6.8]	-			
**Health literacy**							**P**	**S**	**E**	
	**Inadequate** (N=26)	10	-1.3 (1.4)	16	4.3 (1.2)	5.6 [1.9 – 9.2]	0.189	0.994	0.733	
	**Problematic P** (N=66)	36	0.3 (0.8)	30	3.1 (0.9)	2.7 [0.5 – 5.0]	-	0.060	0.299	
	**Sufficient S** (N=75)	37	-1.5 (0.8)	38	4.1 (0.8)	5.6 [3.6 – 7.6]	-	-	0.649	
	**Excellent E** (N=32)	16	0.0 (1.1)	16	4.7 (1.2)	4.8 [1.6 – 7.9]	-	-	-	
**Tinnitus perception**							**Atonal**	**T/A**		
	**Tonal** (N=140)	69	-0.4 (0.6)	71	3.7 (0.6)	4.1 [2.5 – 5.6]	0.356	0.406		
	**Atonal** (N=43)	22	-0.7 (1.0)	21	4.8 (1.0)	5.5 [2.8 – 8.2]	-	0.868		
	**Tonal/Atonal** (N=16)	8	-1.8 (1.6)	8	4.1 (1.5)	5.9 [1.7 – 10.2]	-	-		
**Hearing problems**							**No**			
	**Yes** (N=100)	49	-0.6 (0.7)	51	3.7 (0.7)	4.3 [2.5 – 6.1]	0.715			
	**No** (N=99)	50	-0.6 (0.7)	49	4.1 (0.7)	4.8 [3.0 – 6.6]	-			
**Use of hearing aids**							**No**			
	**Yes** (N=19)	9	0.5 (1.5)	10	3.3 (1.5)	2.8 [-1.3 – 6.9]	0.390			
	**No** (N=180)	90	-0.7 (0.5)	90	4.0 (0.6)	4.7 [3.4 – 6.0]	-			
**Sensitivity to noise**							**R**	**S**	**U**	**A**
	**Never** (N=16)	10	-0.3 (1.4)	6	5.9 (1.8)	6.1 [1.7 – 10.6]	0.28	0.50	0.88	0.43
	**Rarely R** (N=43)	23	-0.2 (1.0)	20	3.1 (1.1)	3.2 [0.4 – 6.0]	-	0.48	0.20	0.90
	**Sometimes S** (N=81)	37	-0.3 (0.8)	44	4.2 (0.8)	4.5 [2.4 – 6.5]	-	-	0.45	0.73
	**Usually yes U** (N=45)	23	-1.8 (1.0)	22	3.9 (1.0)	5.8 [3.0 – 8.5]	-	-	-	0.42
	**Always A** (N=14)	6	0.0 (1.8)	8	3.5 (1.5)	3.6 [-1.1 – 8.3]	-	-	-	-
**Number of tinnitus treatments**							**2–4**	**1**	**0**	**NS**
	**≥ 5** (N=21)	14	-2.5 (1.2)	7	4.4 (1.8)	6.9 [2.7 – 11.1]	0.21	0.15	0.46	0.28
	**2–4** (N=56)	24	0.1 (0.9)	32	4.0 (0.8)	3.9 [1.5 – 6.3]	-	0.78	0.45	0.59
	**1** (N=51)	24	0.3 (0.9)	27	3.7 (0.9)	3.4 [0.9 – 5.9]	-	-	0.31	0.66
	**0** (N=67)	34	-1.0 (0.8)	33	4.2 (0.8)	5.1 [2.9 – 7.3]	-	-	-	0.43
	**Not specified** (N=4)	3	-1.2 (2.5)	1	0.0 (4.2)	1.2 [-8.4 – 10.7]	-	-	-	-

Page 1 of 2

Table S7 (contd). Primary endpoint analysis (Mini-TQ-12 score) with jump-to-reference imputation of missing t1 data, comparing diverse subgroups of patients.

Subgroups		Change from t0 to t1				Treatment difference from t0 to t1 (control vs. intervention) EMM [1] [95% CI]	Pairwise subgroup comparison of treatment difference *p*-value [2]		
		Control group		Intervention group					
		n	EMM [1] (SE)	n	EMM [1] (SE)				
	Total (N = 199)	99	-0.6 (0.5)	100	3.9 (0.5)	4.5 [3.3 – 5.8]			
**Tinnitus loudness**							**Medium**		**High**
	**Low** (N=48)	20	0.4 (1.1)	28	4.1 (1.0)	3.8 [1.1 – 6.5]	0.990		0.179
	**Medium** (N=90)	43	0.0 (0.7)	47	3.7 (0.7)	3.8 [1.9 – 5.6]	-		0.102
	**High** (N=61)	36	-1.7 (0.8)	25	4.5 (1.0)	6.2 [3.9 – 8.5]	-		-
**Tinnitus annoyance**							**ML**	**None**	**NS**
	**Strong** (N=73)	40	-1.4 (0.8)	33	3.7 (0.9)	5.1 [3.0 – 7.2]	0.532	N/A	N/A
	**Moderate to low** (N=123)	56	-0.1 (0.7)	67	4.1 (0.6)	4.3 [2.7 – 5.8]	-	N/A	N/A
	**None** (N=1)	1	2.5 (4.3)	0	N/A	N/A	-	-	N/A
	**Not specified** (N=2)	2	-0.3 (4.0)	0	N/A	N/A	-	-	-
**Problems with language comprehension**							**ML**	**None**	**NS**
	**Very strong to strong** (N=45)	26	-1.7 (0.9)	19	4.8 (1.0)	6.6 [4.0 – 9.1]	0.139	0.112	N/A
	**Moderate to low** (N=102)	49	-0.5 (0.7)	53	3.7 (0.7)	4.2 [2.4 – 6.0]	-	0.746	N/A
	**None** (N=51)	24	0.4 (1.0)	27	4.1 (0.9)	3.7 [1.2 – 6.2]	-	-	N/A
	**Not specified** (N=1)	0	N/A	1	-1.5 (5.3)	N/A	-	-	-
**Medication intake against tinnitus at t0**									**No**
	**Yes** (N=25)	12	-0.8 (1.4)	13	5.5 (1.4)	6.2 [2.5 – 10.0]			0.329
	**No** (N=174)	87	-0.5 (0.5)	87	3.7 (0.6)	4.3 [3.0 – 6.6]			-
**Other forms of tinnitus therapy at t0**									**No**
	**Yes** (N=5)	2	-1.9 (3.4)	3	6.4 (3.0)	8.4 [-0.7 – 17.4]			0.399
	**No** (N=194)	97	-0.5 (0.5)	97	3.9 (0.5)	4.4 [3.1 – 5.7]			-

[1] Calculated using a linear mixed regression model with the change in the Mini-TQ-12 score as the dependent variable, intervention as a dummy variable (0 = control group, 1 = intervention group), baseline value and age as continuous covariates, sex as a dummy variable (0 = male, 1 = female), subgroup characteristics as a categorical variable (reference: first subgroup), the interaction between the intervention variable and the subgroup characteristic as well as the ENT practice IDs as random model constants. [2] *p*-value based on the non-directional hypothesis of a difference in the intervention variable between the considered subgroups and calculated based on the corresponding regression coefficient of the interaction term. Abbreviations: CI, confidence interval; EMM, estimated marginal mean; ENT, Ear, Nose and Throat medical specialty; FAS, full analysis set; N/A, not applicable; N, number of patients per subgroup or in total; n, number of patients per analysis group; SE, standard error; t0, baseline (study start); t1, endline (10 weeks after study start).

Table S8. Secondary endpoint analysis (BVB-2000 score) as single-point measurement at t1 with predictive mean matching imputation of missing values, comparing diverse subgroups of patients.

Subgroups		Single-point measurement at t1: Control group n	Control group EMM [1] (SE)	Intervention group n	Intervention group EMM [1] (SE)	Treatment difference at t1 (control vs. intervention) EMM [1] [95% CI]	Pairwise subgroup comparison of treatment difference *p*-value [2]			
	Total (N = 199)	99	4.1 (0.1)	100	4.5 (0.1)	0.5 [0.3 – 0.7]				
**Age (years)**							**49–58**	**58-82**		
	**18–49** (N=69)	27	3.9 (0.1)	42	4.4 (0.1)	0.4 [0.1 – 0.8]	0.315	0.964		
	**49–58** (N=66)	37	3.9 (0.1)	29	4.7 (0.1)	0.7 [0.4 – 1.1]	-	0.298		
	**58–82** (N=64)	35	4.0 (0.1)	29	4.4 (0.2)	0.4 [0.0 – 0.8]	-	-		
**Sex**							**Female**			
	**Male** (N=101)	45	3.9 (0.1)	56	4.5 (0.1)	0.6 [0.3 – 0.9]	0.569			
	**Female** (N=98)	54	4.0 (0.1)	44	4.5 (0.1)	0.5 [0.3 – 0.7]	-			
**Health literacy**							**P**	**S**	**E**	
	**Inadequate** (N=26)	10	3.8 (0.2)	16	4.6 (0.2)	0.8 [0.2 – 1.4]	0.414	0.099	0.899	
	**Problematic P** (N=66)	36	3.9 (0.1)	30	4.4 (0.1)	0.6 [0.2 – 0.9]	-	0.253	0.462	
	**Sufficient S** (N=75)	37	4.0 (0.1)	38	4.3 (0.1)	0.3 [-0.1 – 0.6]	-	-	0.102	
	**Excellent E** (N=32)	16	4.1 (0.2)	16	4.9 (0.2)	0.8 [0.3 – 1.3]	-	-	-	
**Tinnitus perception**							**Atonal**	**T/A**		
	**Tonal** (N=140)	69	3.9 (0.1)	71	4.5 (0.1)	0.6 [0.4 – 0.9]	0.166	0.584		
	**Atonal** (N=43)	22	4.0 (0.2)	21	4.3 (0.2)	0.2 [-0.2 – 0.7]	-	0.704		
	**Tonal/Atonal** (N=16)	8	3.9 (0.3)	8	4.3 (0.2)	0.4 [-0.3 – 1.1]	-	-		
**Hearing problems**							**No**			
	**Yes** (N=100)	49	3.8 (0.1)	51	4.4 (0.1)	0.6 [0.3 – 0.9]	0.523			
	**No** (N=99)	50	4.1 (0.1)	49	4.6 (0.1)	0.5 [0.2 – 0.8]	-			
**Use of hearing aids**							**No**			
	**Yes** (N=19)	9	3.6 (0.2)	10	4.0 (0.2)	0.4 [-0.3 – 1.0]	0.631			
	**No** (N=180)	90	4.0 (0.1)	90	4.5 (0.1)	0.5 [0.3 – 0.7]	-			
**Sensitivity to noise**							**R**	**S**	**U**	**A**
	**Never** (N=16)	10	3.7 (0.2)	6	4.7 (0.3)	1.0 [0.2 – 1.7]	0.11	0.44	0.27	0.91
	**Rarely R** (N=43)	23	4.2 (0.2)	20	4.4 (0.2)	0.2 [-0.2 – 0.7]	-	0.17	0.46	0.13
	**Sometimes S** (N=81)	37	3.9 (0.1)	44	4.5 (0.1)	0.6 [0.3 – 1.0]	-	-	0.57	0.52
	**Usually yes U** (N=45)	23	4.1 (0.2)	22	4.5 (0.2)	0.5 [0.0 – 0.9]	-	-	-	0.33
	**Always A** (N=14)	6	3.4 (0.3)	8	4.3 (0.2)	0.9 [0.1 – 1.6]	-	-	-	-
**Number of tinnitus treatments**							**2–4**	**1**	**0**	**NS**
	**≥ 5** (N=21)	14	3.9 (0.2)	7	4.3 (0.1)	0.5 [-0.2 – 1.2]	0.75	0.73	0.82	0.98
	**2–4** (N=56)	24	3.9 (0.2)	32	4.5 (0.1)	0.6 [0.2 – 1.0]	-	0.36	0.88	0.89
	**1** (N=51)	24	4.0 (0.2)	27	4.4 (0.1)	0.3 [-0.1 – 0.8]	-	-	0.41	0.85
	**0** (N=67)	34	4.1 (0.1)	33	4.6 (0.1)	0.6 [0.2 – 0.9]	-	-	-	0.93
	**Not specified** (N=4)	3	3.5 (0.4)	1	4.0 (0.1)	0.5 [-1.1 – 2.1]	-	-	-	-

Page 1 of 2

Table S8 (contd). Secondary endpoint analysis (BVB-2000 score) as single-point measurement at t1 with predictive mean matching imputation of missing values, comparing diverse subgroups of patients.

**Subgroups**		**Single-point measurement at t1**				**Treatment difference at t1 (control vs. intervention) EMM [1] [95% CI]**	**Pairwise subgroup comparison of treatment difference *p*-value [2]**		
		**Control group**		**Intervention group**					
		**n**	**EMM [1] (SE)**	**n**	**EMM [1] (SE)**				
	Total (N = 199)	99	4.1 (0.1)	100	4.5 (0.1)	0.5 [0.3 – 0.7]			
**Tinnitus loudness**							**Medium**		**High**
	**Low** (N=48)	20	3.9 (0.2)	28	4.4 (0.2)	0.5 [0.1 – 0.9]	0.646		0.590
	**Medium** (N=90)	43	4.1 (0.1)	47	4.5 (0.1)	0.4 [0.1 – 0.7]	-		0.252
	**High** (N=61)	36	3.8 (0.1)	25	4.5 (0.1)	0.7 [0.3 – 1.0]	-		-
**Tinnitus annoyance**							**ML**	**None**	**NS**
	**Strong** (N=73)	40	3.8 (0.1)	33	4.5 (0.1)	0.7 [0.3 – 1.0]	0.176	N/A	N/A
	**Moderate to low** (N=123)	56	4.0 (0.1)	67	4.4 (0.1)	0.4 [0.1 – 0.6]	-	N/A	N/A
	**None** (N=1)	1	3.9 (0.7)	0	N/A	N/A	-	-	N/A
	**Not specified** (N=2)	2	2.8 (0.6)	0	N/A	N/A	-	-	-
**Problems with language comprehension**							**ML**	**None**	**NS**
	**Very strong to strong** (N=45)	26	3.7 (0.1)	19	4.4 (0.2)	0.7 [0.3 – 1.1]	0.639	0.200	N/A
	**Moderate to low** (N=102)	49	3.9 (0.1)	53	4.5 (0.1)	0.6 [0.3 – 0.9]	-	0.314	N/A
	**None** (N=51)	24	4.3 (0.2)	27	4.6 (0.1)	0.3 [-0.1 – 0.7]	-	-	N/A
	**Not specified** (N=1)	0	N/A	1	4.4 (0.9)	N/A	-	-	-
**Medication intake against tinnitus at t0**									**No**
	**Yes** (N=25)	12	3.7 (0.2)	13	4.3 (0.2)	0.6 [0.0 – 1.1]			0.063
	**No** (N=174)	87	4.0 (0.1)	87	4.0 (0.1)	0.0 [-0.2 – 0.2]			-
**Other forms of tinnitus therapy at t0**									**No**
	**Yes** (N=5)	2	4.2 (0.5)	3	4.1 (0.4)	-0.1 [-1.4 – 1.0]			0.767
	**No** (N=194)	97	4.0 (0.1)	97	4.0 (0.1)	0.1 [-0.1 – 0.3]			-

[1] Calculated using a linear mixed regression model with the change in the BVB-2000 score at t1 as the dependent variable, intervention as a dummy variable (0 = control group, 1 = intervention group), age as a continuous covariate, sex as a dummy variable (0 = male, 1 = female), subgroup characteristics as a categorical variable (reference: first subgroup), the interaction between the intervention variable and the subgroup characteristic as well as the ENT practice IDs as random model constants.
[2] *p*-value based on the non-directional hypothesis of a difference in the intervention variable between the considered subgroups and calculated based on the corresponding regression coefficient of the interaction term. Abbreviations: CI, confidence interval; EMM, estimated marginal mean; ENT, Ear, Nose and Throat medical specialty; FAS, full analysis set; N/A, not applicable; N, number of patients per subgroup or in total; n, number of patients per analysis group; SE, standard error; t0, baseline (study start); t1, endline (10 weeks after study start).
